# Novel Health Information Technology Tool Use by Adult Patients Undergoing Allogeneic Hematopoietic Cell Transplantation: Longitudinal Quantitative and Qualitative Patient-Reported Outcomes

**DOI:** 10.1200/CCI.17.00110

**Published:** 2018-01-11

**Authors:** Lyndsey Runaas, Flora Hoodin, Anna Munaco, Alex Fauer, Roshun Sankaran, Tracey Churay, Saara Mohammed, Sajjad Seyedsalehi, Grant Chappell, Noelle Carlozzi, Michael D. Fetters, Rachel Kentor, Leah McDiarmid, Kristina Brookshire, Casiana Warfield, Michelle Byrd, Sharon Kaziunas, Molly Maher, John Magenau, Larry An, Amy Cohn, David A. Hanauer, Sung Won Choi

**Affiliations:** **Lyndsey Runaas**, **Flora Hoodin**, **Tracey Churay**, **Saara Mohammed**, **Sajjad Seyedsalehi**, **Grant Chappell**, **Noelle Carlozzi**, **Michael D. Fetters**, **Sharon Kaziunas**, **Molly Maher**, **John Magenau, Larry An**, **David A. Hanauer**, and **Sung Won Choi**, Michigan Medicine; **Anna Munaco**, **Alex Fauer**, **Roshun Sankaran**, and **Amy Cohn**, University of Michigan College of Engineering, Ann Arbor; and **Flora Hoodin**, **Rachel Kentor**, **Leah McDiarmid**, **Kristina Brookshire**, **Casiana Warfield**, and **Michelle Byrd**, Eastern Michigan University, Ypsilanti, MI

## Abstract

**Purpose:**

Health information technology (IT) is an ideal medium to improve the delivery of patient-centered care and increase patient engagement. Health IT interventions should be designed with the end user in mind and be specific to the needs of a given population. Hematopoietic cell transplantation (HCT), commonly referred to as blood and marrow transplantation (BMT), is a prime example of a complex medical procedure where patient-caregiver-provider engagement is central to a safe and successful outcome. We have previously reported on the design and development of an HCT-specific health IT tool, BMT Roadmap.

**Methods:**

This study highlights longitudinal quantitative and qualitative patient-reported outcomes (PROs) in 20 adult patients undergoing allogeneic HCT. Patients completed PROs at three time points (baseline, day 30 post-HTC, and day 100 post-HCT) and provided weekly qualitative data through semistructured interviews while using BMT Roadmap.

**Results:**

The mean hospital stay was 23.3 days (range, 17 to 37 days), and patients had access to BMT Roadmap for a mean of 21.3 days (range, 15 to 37 days). The total time spent on BMT Roadmap ranged from 0 to 139 minutes per patient, with a mean of 55 minutes (standard deviation, 47.6 minutes). We found that patients readily engaged with the tool and completed qualitative interviews and quantitative PROs. The Patient Activation Measure, a validated measure of patient engagement, increased for patients from baseline to discharge and day 100. Activation was significantly and negatively correlated with depression and anxiety PROs at discharge, suggesting that this may be an important time point for intervention.

**Conclusion:**

Given the feasibility and promising results reported in this study, next steps include expanding our current health IT platform and implementing a randomized trial to assess the impact of BMT Roadmap on critical PROs.

## INTRODUCTION

Hematopoietic cell transplantation (HCT), commonly referred to as blood and marrow transplantation (BMT), is a complex procedure wherein patients accept significant risks in return for the possibility of cure.^1^ These risks are not time limited or static, but instead are variable in course and severity.^2^ Given this, HCT patients face numerous mental, physical, and emotional challenges across the HCT trajectory^3-5^ and represent one of the most critically ill cancer populations.^6^ The best model of care for such medically complex, and often chronically ill patients, is one that supports patient-centered health care by integrating the use of patient-reported outcomes (PROs).^7,8^ Moreover, patients who are well informed and engaged with their care experience have improved health and psychosocial outcomes.^9,10^

Despite this knowledge, strategies to assess the utility of PROs in clinical practice remain underdeveloped.^7^ Advances in health information technology (IT) offer opportunities to capture PROs and thereby foster patient-provider engagement, which may lead to improved clinical processes and outcomes. We have previously reported on our work examining the information needs of HCT patients, caregivers, and health care providers (“providers”).^11,12^ We identified three stages of the patient-caregiver experience that could be supported by a health IT system, with the goal of enhancing patient, caregiver, and provider engagement. These three stages include the following: (1) navigating the health system and learning to communicate effectively with the health care team; (2) managing daily challenges of caregiving; and (3) transitioning from inpatient care to long-term outpatient management.^11,12^ These findings resulted in the development of a patient-centric health IT tool designed to meet the specific information needs of this patient population.^13,14^

BMT Roadmap is a Web-based IT application on a portable tablet (Apple iPad), which integrates patient-specific health information in real time from the electronic medical record (Epic).^13^ It includes modules for laboratory results, medications with plain language summaries, clinical trial summaries and consent documents, a yearbook-style provider directory, phases of care (trajectory of HCT), and an interactive discharge checklist. We have previously reported on the components of BMT Roadmap^13,14^ as well as our qualitative results on the use of this tool by caregivers of pediatric HCT patients.^15-18^ Herein, we report our quantitative findings of BMT Roadmap use among adult allogeneic HCT recipients, with specific investigation of the impact of the tool on patient engagement through PRO data capture. We also integrate these findings with qualitative data provided by the participants through semistructured interviews. The primary objective of this study was to assess the feasibility of implementing BMT Roadmap in a high-risk allogeneic HCT patient population and capturing PROs longitudinally across a care continuum.

## METHODS

### Procedures

This study was approved by the University of Michigan Institutional Review Board (no. HUM00107014) and registered on ClinicalTrials.gov (identifier NCT03161665). Twenty consecutive allogeneic HCT transplant candidates were recruited by HCT registered nurse coordinators and physicians during the pretransplant work-up stage in the ambulatory care setting before admission to the inpatient HCT unit. All 20 recruited participants met the following eligibility criteria: age ≥ 21 years, first allogeneic HCT, English proficiency, willingness and ability to provide informed consent, and willingness to adhere to study procedures and reporting requirements. After informed consent was obtained, a research coordinator provided a one-on-one, live demo tutorial on use of the tool. Participants were instructed to use the device freely throughout their hospital stay.

### PROs

Having met our primary objective of establishing the feasibility of participant enrollment per the a priori design of the study, we report herein on longitudinally collected, quantitative PROs measured at three time points: admission (baseline), discharge, and day 100 post-HCT. Day 100 post-HCT is considered an important turning point or milestone, reflecting a major transition to recovery following a vulnerable period of intense adverse effects from the procedure itself. The PROs included usefulness (Perceived Usefulness),^19^ ease of use (Perceived Ease of Use),^19^ activation (Patient Activation Measure),^20,21^ depression (depression subscale on Profile of Mood States),^22^ anxiety (State Trait Anxiety Inventory),^23^ overall distress (Profile of Mood States total score),^22^ distress related to transplant (Impact of Events Scale-Revised),^24^ and cancer-related distress (Cancer and Treatment Distress).^25^ Psychometric properties of the PROs are listed in Table 1.

**Table 1.**
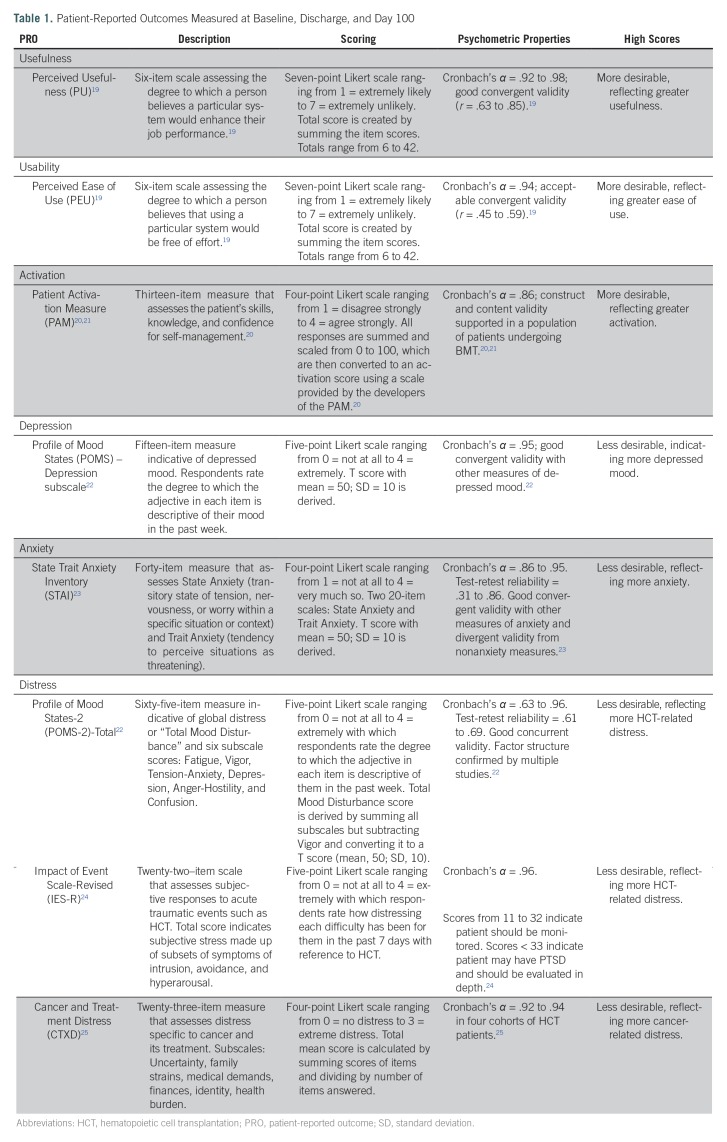
Patient-Reported Outcomes Measured at Baseline, Discharge, and Day 100

### Data Analysis

**Fig 1.**
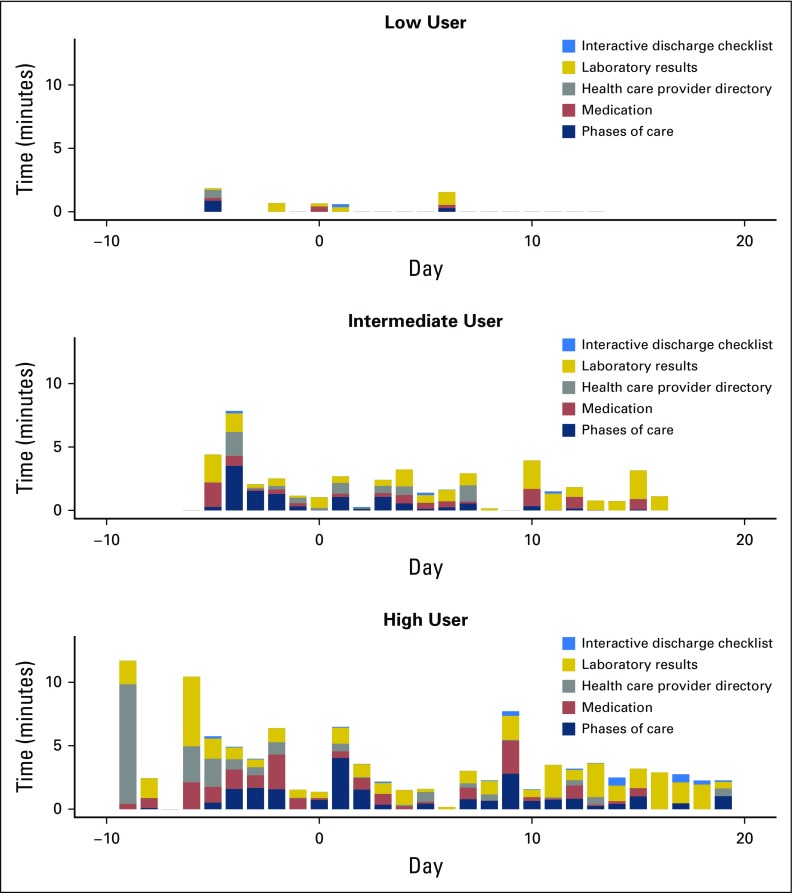
BMT Roadmap use by tertiles. BMT, blood and marrow transplantation.

***Quantitative data.*** PROs were administered electronically on an Apple iPad tablet using the Qualtrics application, which is a secure online Health Insurance Portability and Accountability Act–compliant platform for administering surveys (www.Qualtrics.com). Time stamps of BMT Roadmap log-in and usage were automatically recorded. Statistical analyses were conducted using Statistical Package for Social Sciences (SPSS) Version 24.^26^ Demographic data and clinical characteristics of the study were calculated. Descriptive statistics were also calculated for utilization data and each PRO. Tertiles on the basis of frequency of total minutes of utilization were derived as follows: low (< 7 minutes: 30th percentile), intermediate (7 to 55.5 minutes: 30th to 65th percentile), and high (> 56 minutes: > 65th percentile; Fig 1). Missing data (individual unanswered items) within the standardized measures were imputed using individualized mean substitutions within in each measure. Repeated measures analysis of variance was used to determine change in PROs over time. At each time point, analysis of covariance (ANCOVA) was used to examine effects of utilization group on patient activation while accounting for variance as the result of age. Pearson correlations were used to determine the significance of association among demographic data (age, education), medical variables (length of hospital stay, cell dose), and PROs.

***Qualitative data.*** Semistructured interviews were performed weekly during the participant’s inpatient stay to assess their qualitative interaction with BMT Roadmap, and at days 30 and 100 post-transplant, as previously described.^17^ Weekly interviews were conducted to check in with patients given the feasibility nature of this study, particularly to resolve any technical difficulties, but also to mitigate problems and repair them in real time. Briefly, all qualitative interviews were audio-recorded with permission, de-identified, professionally transcribed (Babbletype, Philadelphia, PA), and entered and coded in NVivo Pro 11. Transcriptions were reviewed and coded by a minimum of two research assistants until consensus was achieved. To ensure the quality and robustness of our findings, we performed interobserver reliability analysis on 10% of the coded interviews and demonstrated excellent reliability (κ = 0.96).^27^ A qualitative constant comparison of the data was conducted using a consistent set of codes (deductive approach).^28^ The coding structure was refined through iterative cycles of coding and data collection, and new emerging codes were also included (inductive approach).^17,28^

## RESULTS

### Demographic Data

Twenty HCT patients undergoing first-time allogeneic HCT enrolled in the study between January and June 2016. All patients approached for the study enrolled and provided consent; no one declined. As shown in Table 2, the mean age of participants was 53 years (range, 27 to 71 years). The sample was predominantly male (n = 15), white (n = 18), and most participants had at least a high school diploma or higher education (n = 16). The indication for HCT was malignancy (n = 20). The majority of patients had an intermediate- to high-risk HCT comorbidity index^29^ (n = 14) and received an unrelated donor HCT (n = 11) from peripheral blood stem cell donors (n = 14).

**Table 2.**
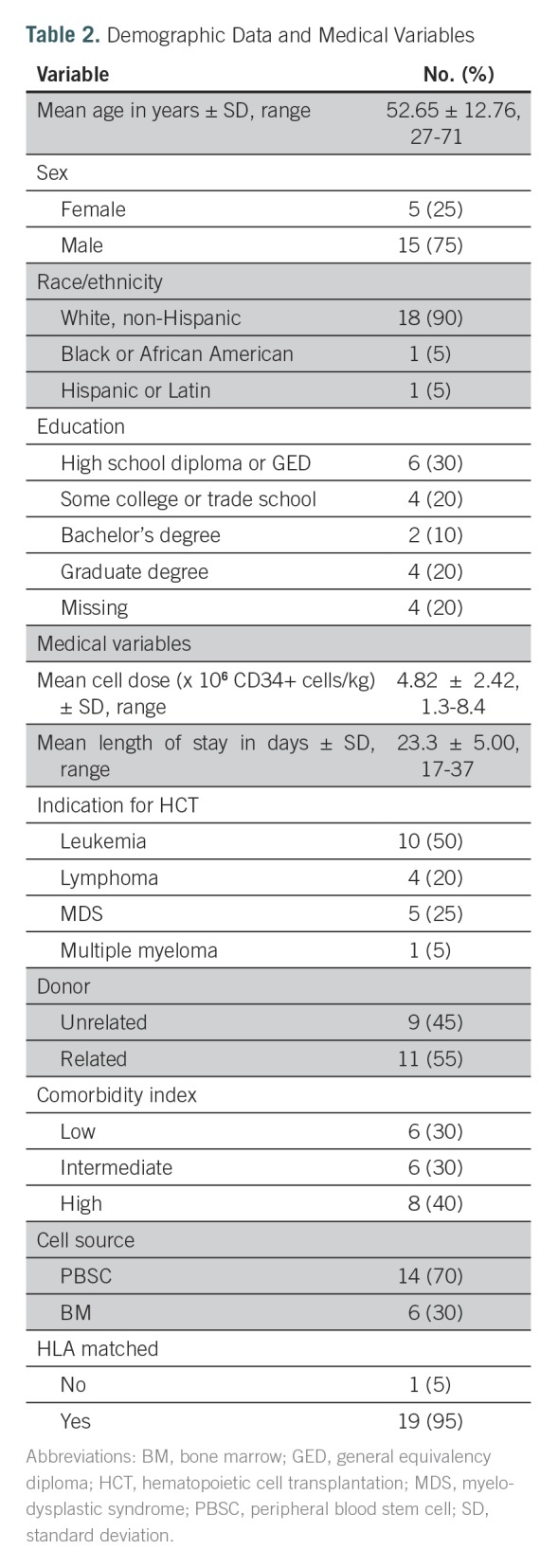
Demographic Data and Medical Variables

The mean hospital stay for all study participants was 23.3 days (range, 17 to 37 days), and they had access to BMT Roadmap for a mean of 21.3 days (range, 15 to 37 days). The total time spent on BMT Roadmap ranged from 0 to 139 minutes per patient, with a mean of 55 minutes (standard deviation [SD], 47.6 minutes). Participants logged in at least once per day for a mean of 7.6 days (SD, 6.3 days). Participants spent the majority of their time in the laboratory module, followed by phases of care and medications. The least used module was the discharge checklist (Table 3).

**Table 3.**
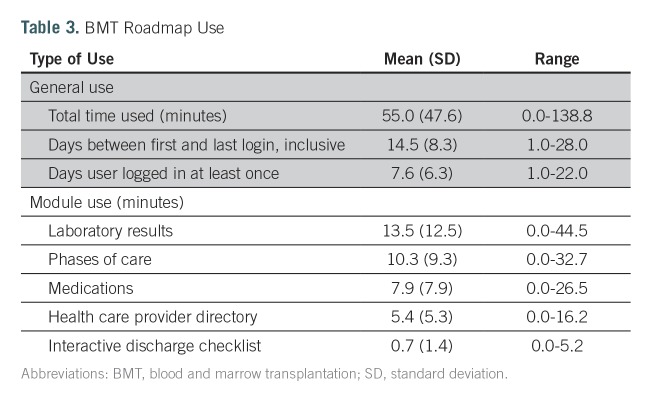
BMT Roadmap Use

### PRO Measures

Eleven participants completed all of the PROs at each time point. Four patients did not complete baseline PROs as the result of technical error; two patients declined to complete the day 30 and day 100 PROs; and three patients died after discharge and did not complete day 100 PROs.

### Perceived Usefulness and Perceived Ease of Use

Both Perceived Usefulness and Perceived Ease of Use showed high internal consistency (Cronbach’s alpha = 0.98). Perceived Usefulness and Perceived Ease of Use scores were in the intermediate range and did not change significantly from baseline to discharge to day 100 (Table 4).

**Table 4.**
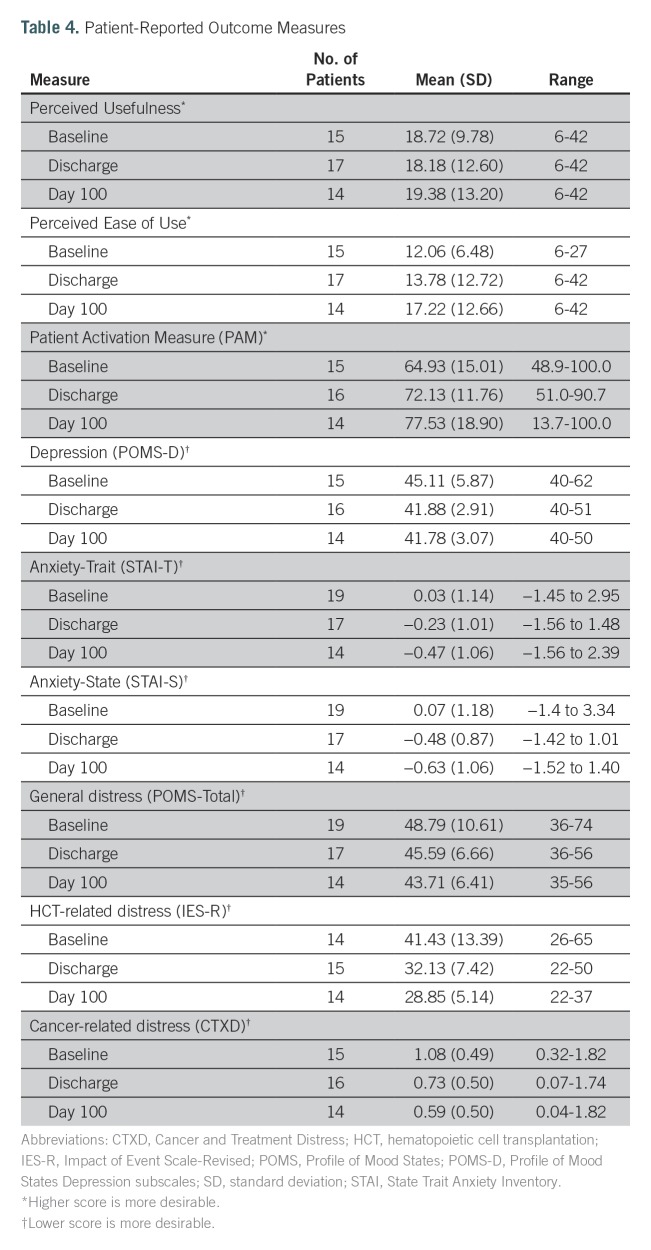
Patient-Reported Outcome Measures

### Patient Activation Measure

Mean Patient Activation Measure (PAM) scores increased over the three time points (Table 4). At day 100, activation was inversely related to length of stay, ie, the more days spent as an inpatient, the lower the PAM score (*r* = −0.52, *P* = .05). The percent of the sample who scored at PAM level 4, the highest level of activation,^21,22^ was 33.3% at baseline, 62.5% at discharge, and 57.1% at day 100. Among the 11 participants who completed the PAM at all three time points, repeated measures analysis of variance showed that activation significantly increased over time, with scores of 67.6 (SD, 16.65) at baseline, 72.8 (SD, 13.62) at discharge, and 77.7 (SD, 19.85) at day 100 (*F* = 5.64, *P* = .04).

Interestingly, we found that PAM scores were not linearly related to the total time BMT Roadmap was used. We then explored activation stratified by tertiles of total minutes of use. At discharge and day 100, the intermediate users showed a trend toward increased activation versus the lowest and highest users (*F* = 3.52, *P* = .06 and *F* = 3.62, *P* = .06, respectively), suggesting a quadratic or nonlinear relationship between the PAM and minutes of use.

As a result of the potential confounding effect of patient age on use of mobile technology, we then compared activation levels of intermediate users compared with all others, controlling for age in separate ANCOVAs at each time point. ANCOVAs revealed that at day 100, intermediate users were significantly more activated than low and high users, with mean activation scores of 99.8, 67.7, and 73.5, respectively (*F* [2,10] = 5.29, *P* = .03; partial η2 = 0.51).

### Mental Health PROs

Measures of depression (Profile of Mood States-Depression), trait and state anxiety (State Trait Anxiety Inventory), global distress (Profile of Mood States-Total), HCT-related distress (Impact of Events Scale-Revised), and Cancer and Treatment Distress were assessed to determine the effects of potential confounders on utilization and/or activation. We found that use was not significantly correlated with any of the mental health measures at any of the time points, suggesting that these variables did not confound utilization nor did utilization affect the mental health variables. However, the measures themselves were intercorrelated (Table 5). Notably, at baseline and discharge, depression correlated positively with state anxiety (*r* = 0.71, *P* = .001 and *r* = 0.81, *P* = .001, respectively), trait anxiety (*r* = 0.78, *P* = .001 and *r* = 0.71, *P* = .01, respectively), global distress (*r* = 0.80, *P* = .001 and *r* = 0.63, *P* = .01, respectively), and cancer-related distress (*r* = 0.68, *P* = .01 and *r* = 0.61, *P* = .05, respectively). By day 100, these relationships were attenuated, such that depression was associated to a significant degree only with state anxiety and global distress (Table 5).

**Table 5.**
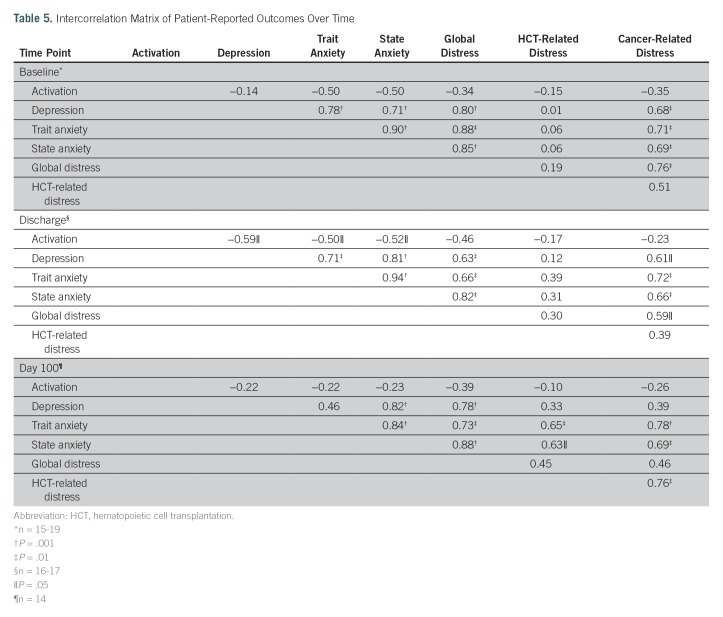
Intercorrelation Matrix of Patient-Reported Outcomes Over Time

Activation was not associated with any of the mental health PROs at baseline or day 100 (ie, the two time points that represent time periods of no BMT Roadmap use). Interestingly, at discharge, activation correlated significantly and negatively with depression (*r* = −0.59, *P* = .04), trait anxiety (*r* = −0.52, *P* = .05), and state anxiety (*r* = −0.52, *P* = .05), suggesting that greater activation was associated with less depression and anxiety.

Depression and HCT-related distress (Impact of Event Scale-Revised) decreased significantly from baseline to discharge to day 100 post-HCT (*F* = 7.83, *P* = .01 and *F* = 5.36, *P* = .02, respectively). Similar trends were observed for state anxiety (*F* = 3.59, *P* = .08) and cancer-related distress (*F* = 4.26, *P* = .07).

Age, length of stay, and cell dose were not correlated with any mental health PROs at any time point. However, more educated patients showed more state anxiety at baseline and discharge (*r* = 0.62, *P* = .01 and *r* = 0.54, *P* = .03, respectively). In addition, at discharge, the patients who were more educated were more depressed (*r* = 0.62, *P* = .01) and exhibited a trend toward more overall emotional distress (Profile of Mood States-Total: *r* = 0.47, *P* = .07). By day 100, education was not correlated with any of the mental health PROs.

### Integration of Quantitative PROs With Qualitative Findings

Participants were engaged in weekly semistructured interviews (Data Supplement). The five themes that emerged from weekly semistructured interviews in adult HCT patients were consistent with our prior findings in caregivers of pediatric HCT patients, which included the following: (1) emotional impact of the HCT process itself; (2) critical importance of communication among patients, caregivers, and providers; (3) ways in which BMT Roadmap was helpful in the inpatient setting; (4) suggestions for improvement of BMT Roadmap; and (5) other strategies for organization and management of complex health care needs that could be incorporated into BMT Roadmap.^17^ We provide a summary of representative qualitative data from adult HCT patients that are integrated with each PRO (Data Supplement).

## DISCUSSION

There is a growing expectation that safe health care across the continuum must be organized using patient-centered systems.^30^ To achieve this, an integrated, multidisciplinary approach is needed to facilitate patient communication, collaboration, and efficiency.^31^ On the basis of user-centered design techniques, our multidisciplinary team developed a novel health IT tool for HCT patients to use during their hospital admission.^12,14^ We examined the views and perspectives of adult HCT recipients interacting with BMT Roadmap through qualitative interviews and quantitative PROs. To our knowledge, this is the first report of adult HCT patients interacting with a personalized health IT tool throughout their transplantation admission.

A central finding of our work was that patient activation increased across time (ie, baseline, discharge, and day 100). Importantly, increased activation at discharge was shown to be associated with reduced depression and anxiety. This suggests that activation may be a useful PRO measure for future HCT studies, as described in earlier studies in the pediatric HCT setting.^32,33^ Among adult HCT recipients, the burden of unmet mental health needs is high.^34^ Studies have shown that depression is associated with nonadherence to the post-HCT regimen, increased hospital length of stay, greater mortality, and increased suicidal ideation.^35,36^ Accordingly, efforts to provide patients with an interactive health IT avenue of communication with providers may improve activation as well as affect mental health variables.

Intriguingly, in our study, activation was not linearly related to minutes of use of the tool; patients who used the tool more were not necessarily more activated. Instead, we found that intermediate users had the highest levels of activation versus either low or high users. Although it is difficult to draw any specific conclusions from this finding given our nonrandomized data and small sample size, it suggests that there may be a sweet spot in terms of information utilization. It is possible that the tool was not effectively tailored for low or high users. For example, high users may have required even more information related to their health care. BMT Roadmap may not have adequately provided the information they needed to help them adopt new health behaviors, become more goal oriented, or display positive emotions. We were encouraged that participants were willing to complete PROs on the BMT Roadmap platform and share their experiences through qualitative interviews, despite being an older population with intermediate to high HCT comorbidity index.^29^ Accordingly, tailored information delivery according to PRO output may improve usefulness and usability of health IT tools and should be examined in future studies.^37^

Our preliminary findings reported herein are encouraging and provide a framework for future work using larger, more heterogeneous patient populations. A limitation of our study was related to technological barriers that participants experienced. These barriers, primarily related to logging on to the secure wireless network or being timed out and having to relog on to the program, are well-reported challenges.^37^ These barriers are likely to be overcome in future iterations of the IT tool, but will certainly need to be taken into account when other tools are developed. In part, these technical limitations are related to additional security enforced to protect patient privacy. Nonetheless, the strengths of this study include its longitudinal nature from admission through day 100, our rigorous methodology to evaluate patient views and perspectives with repeated qualitative assessments,^17^ and collection of well-validated PROs at three time points across the acute HCT trajectory. In contrast to five other early adopters of acute inpatient portals,^37^ our participants used BMT Roadmap for a longer hospital course and were followed prospectively with qualitative and quantitative PROs for > 100 days. In this study, patients had access to BMT Roadmap for a mean of 21.3 hospital days, which is longer than the duration of use described in other centers with inpatient portals, such as Brigham and Women’s Hospital (Patient-Centered Toolkit), New York Hospital Presbyterian (myNYP.org), Ohio State University Wexner Medical Center (MyChart Bedside), El Camino Hospital Family Medical Officer (iFMO), and Northwestern Memorial Hospital.^36^ This suggests that further inquiry into the benefits of developing department- or disease-specific tools may be beneficial to patients. Such tools may also serve to improve their education and encourage involvement in their own care. Interpretation of our findings herein are further discussed in the Data Supplement.

To our knowledge, no other study has followed a cancer population for this length of time (ie, daily inpatient use during intense HCT therapy), while assessing the utility of a mobile IT application through both quantitative and qualitative PROs to help support the inpatient journey. We have an ongoing study assessing the impact of BMT Roadmap on caregivers of adult HCT patients. Our findings collectively indicate that it is critical to understand user views and perspectives in the design and development of such tools, which we did throughout the design and implementation of this project. Our key end users included pediatric patients and their caregivers (eg, parents, grandparents), as well as adult patients and their caregivers (eg, spouses, adult children). In addition, a collaborative and multidisciplinary team with input from providers, health informaticists, data scientists, computer scientists, engineers, and health communication scientists was critical in the design, implementation, and evaluation of BMT Roadmap. Nonetheless, we recognize the major limitations of our study, which include the nonrandomized, single-center design; small homogeneous sample; and technical barriers. However, given the severely ill nature of HCT patients, follow-up was actually fairly complete and overall retention in the study was high. 

In conclusion, given the promising results reported in this study, our next steps include further iterations of the tool itself, focusing on increased ease of use and dynamism, extending use of the tool to outpatients, as well as conducting a randomized trial to assess whether the BMT Roadmap versus standard inpatient teaching is a primary driver of improved patient activation.
